# Tooth-derived graft for preservation and reconstruction of orofacial bones: A comprehensive review

**DOI:** 10.34172/japid.026.3834

**Published:** 2026-03-16

**Authors:** Mina Shekarian, Pardis Amani Beni, Ghazal Hassanzadehganroudsari, Abbasali Khademi

**Affiliations:** ^1^Dental Research Center, Dental Research Institute, School of Dentistry, Isfahan University of Medical Sciences, Isfahan, Iran; ^2^Student Research Committee, School of Dentistry, Isfahan University of Medical Sciences, Isfahan, Iran; ^3^School of Dentistry, Islamic Azad University of Tehran, Tehran, Iran; ^4^Department of Endodontics, Dental Research Center, Dental Research Institute, School of Dentistry, Isfahan University of Medical Sciences, Iran

**Keywords:** Autografts, Bone regeneration, Demineralized dentin matrix, Platelet-rich fibrin

## Abstract

Bone loss in the craniofacial region can result from various causes, requiring grafting for restoration. Each type of bone graft has advantages and disadvantages. In recent years, an increasing number of clinical research has used extracted teeth as graft biomaterials. This study aimed to outline the use of teeth as bone graft material, describe a general protocol for producing tooth graft material, compare various manufacturing methods, and assess tooth-derived grafts relative to other bone graft materials. The review was based on electronic database searches in Scopus, PubMed, Cochrane, Web of Science, and Google Scholar, using various combinations of keywords such as "autogenous graft," "autologous graft," "human dentin," "dentin matrix," "tooth graft," "demineralized dentin matrix," and related terms applied to titles, abstracts, and keywords. Tooth-derived grafts demonstrate promising outcomes comparable to conventional bone graft materials. However, further research is necessary to standardize protocols and validate clinical applications.

## Introduction

 Bone loss in the craniofacial region can occur for several reasons, including traumatic tooth extraction, pathologies, trauma, or periodontal diseases.^1,2^ Grafting is necessary to restore the lost bone. Various grafting materials are available, classified as autogenous, allogenic, alloplastic, xenogeneic, or engineered personalized grafts.^3,4^

 An ideal bone graft material should have four essential characteristics: osteoconductivity (which functions as a scaffold for bone regeneration), osteoinductivity (which contains growth and regulatory factors that stimulate bone formation), osteogenesis (which comprises cells that promote bone formation), and bone binding (which tightly integrates with bone tissue).^5^ Autogenous bone grafts exhibit all these properties and possess excellent biocompatibility. Therefore, they have long been considered the gold standard for bone grafting.^6^ Nevertheless, several drawbacks are associated with the use of autogenous bone grafts, such as inevitable additional surgeries, resorption, and a limited amount of available graft material.^7^ Other bone graft materials may be inadequate in promoting bone formation because of their manufacturing processes, low porosity, and unfavorable host responses.^8^ In this context, allografts are disadvantaged by their lack of osteoproliferative potential, while xenografts and alloplasts provide only osteoconductivity.^9^

 In recent years, an increasing number of clinical studies have explored the use of extracted teeth as grafting biomaterials due to their biocompatibility, non-immunogenicity, osteoconductivity, osteoinductivity, and osteogenicity.^10^

 The purpose of this study was to outline the use of teeth as bone graft material, describe a general protocol for tooth graft material production, compare various manufacturing methods, and assess tooth-derived grafts in comparison to other bone graft materials.

## Review Design

 This comprehensive narrative review was conducted to synthesize current evidence regarding tooth-derived graft materials, their processing protocols, clinical applications, combination with PRF, and available chairside preparation devices. Although not designed as a formal systematic review, the methodology was structured to ensure transparency, reproducibility, and critical appraisal of the available literature.

## Information Sources and Search Strategy

 An electronic literature search was performed using PubMed, Scopus, Web of Science, and Elsevier databases. The search included articles published up to January 2026. Only studies published in English were considered.

 “Autogen* graft” OR “autolog* graft” AND “human dentin” OR “dentin matrix” OR “dentin graft” OR “ATB” OR “ATG” OR “tooth graft” OR “teeth graft” OR “teeth derived graft” OR “tooth derived graft” OR “autologous tooth bone graft” OR “Autologous Tooth Structure” OR “autogen* tooth bone graft” OR “crush teeth” OR “Autogen* Particulate Dentin” OR “Particulate Dentin” OR “demineralized dentin matrix block” OR “Demineralized Dentin Matrix” OR “DDM” were the keywords used in the titles, abstracts, and keywords of articles with diverse combinations. Reference lists of selected articles were manually screened to identify additional relevant studies.

## Eligibility Criteria

 Studies were considered eligible if they investigated tooth-derived graft materials, including autogenous and processed dentin-based grafts, and reported biological, histological, radiographic, or clinical outcomes related to bone regeneration. Articles examining the combination of dentin grafts with adjunctive biologics such as PRF were also included. In addition, studies describing preparation protocols, demineralization techniques, or chairside processing devices were considered relevant. Only original research articles, including in vitro experiments, animal studies, and human clinical investigations, were eligible for inclusion. Non-English publications, editorials, opinion papers without primary data, conference abstracts lacking full-text availability, and studies not directly related to bone regeneration applications were excluded. Case reports were included only when higher levels of evidence were unavailable and were interpreted with caution due to their inherent methodological limitations.

## Study Selection Process

 Titles and abstracts were screened for relevance. Full-text articles were retrieved for studies meeting the inclusion criteria. Articles that did not meet eligibility requirements were excluded after full-text evaluation.

 Duplicate records were removed before screening. When necessary, disagreements regarding inclusion were resolved through discussion among the authors.

## Data Extraction

 From the included studies, data were systematically extracted regarding study design, sample size, and the specific type of graft material used, including mineralized dentin, partially demineralized dentin, fully demineralized dentin, and enamel-containing grafts. Information on particle size range, demineralization protocols, and processing methods was also recorded. Additionally, the use of adjunctive biologics was documented when applicable. Outcome measures included histomorphometric findings, radiographic bone gain, clinical changes in ridge width and height, and, when reported, implant survival rates. Follow-up duration and any reported complications were also extracted to allow for comprehensive qualitative synthesis and critical interpretation of the available evidence.

## Evidence Classification and Critical Appraisal

 To reduce heterogeneity and avoid pooling evidence at different levels without distinction, studies were categorized into in vitro, animal, and human clinical studies. Within the clinical category, randomized controlled trials, cohort studies, case series, and case reports were considered separately.

 Due to the heterogeneity of study designs and outcomes, a quantitative meta-analysis was not feasible. Therefore, findings were synthesized qualitatively. A formal risk-of-bias tool was not uniformly applicable across all study types; however, methodological limitations such as small sample sizes, short follow-up, lack of control groups, and absence of standardized preparation protocols were critically considered in interpreting the results.

## Device Analysis Approach

 Commercial chairside dentin-processing systems were reviewed based on information available in peer-reviewed publications and publicly accessible technical documentation. No independent experimental comparison was performed in this review. When data were derived from manufacturer reports, this was explicitly acknowledged, and conclusions were interpreted cautiously to avoid promotional bias. At present, no independent head-to-head comparative clinical or histomorphometric studies evaluating these commercial systems exist; consequently, no conclusions regarding relative clinical performance or superiority can be drawn.

## Dentin Structure

 Dentin granules possess substantial osteoconductive capacity, serving as a foundation for the deposition of new bone matrix generated by osteoblasts surrounding the granules. This osteoconductive capacity appears to be independent of factors such as the age, type, and condition of the tooth at the time of extraction.^11^ Moreover, the higher density of tooth substances compared to bone may facilitate a slower resorption rate, which is advantageous for bone healing.^9^

 Dentin consists of 70‒75% inorganic content, 20% organic content, and 10% water, while alveolar bone contains 65% inorganic content, 25% organic content, and 10% water.^12^ Approximately 90% of the organic content in dentin is Type I collagen, which plays a supportive role during bone formation. The remaining 10% consists of non-collagenous proteins (NCPs), including bone morphogenetic protein (BMP), insulin-like growth factor II (IGF-II), and transforming growth factor beta (TGF-β), which promote bone formation.^13^ Among these NCPs, BMP plays a significant role in jawbone repair.^14^

## Production of Tooth Graft Material

 In 2008, an innovative bone graft material, Autogenous Tooth-Derived Graft (AutoDG), was introduced for bone defect regeneration. AutoDG is prepared from extracted teeth and grafted into the same donor patient. The organic portion of AutoDG, containing NCPs, possesses osteoinductive potential, while its inorganic component provides osteoconductive potential. AutoDG can be used in various procedures, including sinus lift, alveolar ridge augmentation, ridge preservation, and guided bone regeneration (GBR).^15^ Clinical studies have demonstrated a negligible level of immune reaction and low risk of infection in the recipient sites of AutoDG grafts.^7,12^

 Kim et al.^12^ developed a technique to produce a bone graft material from autogenous teeth, which involves tooth extraction, removal of soft tissues and contaminants, storage in 75% alcohol in a refrigerator or freezer, crown dissection, dental pulp removal, crushing of the teeth, AutoDG treatment, dehydration, and lyophilization.^12^

 Currently, four devices are available for converting a tooth into bone graft material: Bone Maker, Tooth Transformer, Kometabio, and Vacuasonic. Although these systems share general procedural steps such as cleaning, grinding, and chemical processing, their demineralization protocols, chemical compositions, exposure times, and mechanical parameters differ. Based on currently available literature, there is insufficient independent histological or biochemical characterization to definitively classify the final graft materials produced by these devices into established dentin-derived graft categories. Therefore, the following discussion is limited to protocol-based characteristics rather than verified biological equivalency.

 In the first step, tooth cleaning, there is consensus that, after tooth extraction, caries, restorations, and calculus should be removed. Teeth with root canal therapies can be used in some devices (Table 1). There is controversy over which parts of the tooth should be removed and which can be ground for graft preparation. Some studies have focused on using only the root,^20,21^ while others have utilized both the root and crown portions,^22-24^ as well as dentin alone, dentin with pulp,^25,26^ dentin with enamel,^10,26^ and dentin with cementum.^26,27^

**Table 1:** Features of various tooth graft devices

**Device Name**	**Shape of Produced Material**	**Possibility of Using** **- Endodontically treated teeth** **- Deciduous teeth**	**Composition of Liquids**	**Duration of Treatment**	**Usage**	**Speed**	**Granular Size**
Bone Maker ^16-18^	Granular and block	- Yes - Yes	granular formulation: HCl 0.45 M-H2O2 130 volumes-ethanol 62.6% chloroform 31.3% water 6.1% + washing saline solution; block formulation: HCl 0.56 M-H2O2 120 volumes-ethanol 47.2% chloroform 47.2% water 5.6% + washing saline solution.	Granular: 19 minutes and 50 seconds Block: 35 minutes and 50 seconds	Manual/ Automatic	High - speed	450‒850 μm
Vacuasonic ^17^	Granular and block	- Yes - No information based on articles	0.6 M hydrochloric acid (HCl) + peracetic acid + ethanol + phosphate wash buffer solution	Granular: 30 minutes Block: 2 hours	Manual/ Automatic	No information (High-speed)	450‒850 μm
Kometabio ^16,17^	Granular	- No - No information based on articles	0.5 M NaOH + 30% alcohol + EDTA 10% + PBS	Granular: 20 min Block: -	Manual	High- speed	300‒1200 μm
Tooth Transformer ^17,19^	Granular	- Yes - Yes	0.1 M hydrochloric acid, 10% hydrogen peroxide, and demineralized water as a wash	Granular: 30 min Block: -	Automatic	Low-speed	No information

 Some studies discourage the use of enamel due to the challenge osteoclasts face in breaking down its highly crystalline hydroxyapatite structure, leading to slow resorption and reduced osteoconductivity.^10^ In contrast, some others represented enamel granules, as well as the dentin granules, which have intrinsic osteoconductive properties and can act as a scaffold for new bone matrix deposition.^17^ Nevertheless, the use of enamel in tooth-derived graft materials is still debated, as its highly crystalline structure and slow resorption rate may negatively affect graft integration during the healing period.

 Murata et al.^27^ showed direct bonding between new bone and demineralized cementum, while Schwarz et al.^28^ removed the layer of cementum on the downward side of the root to enhance the fusion between the graft and the defect site. Furthermore, the cementum layer of AutoDG exhibits a highly crystalline structure, which has been suggested to contribute to reduced graft resorption during the healing period, as supported by reports of significantly lower resorption rates compared to autogenous bone.^29^

 While dental pulp contains dental pulp stem cells with regenerative properties similar to those of bone marrow-derived mesenchymal stem cells, most protocols recommend removing this soft tissue before processing the tooth.^30^

 Most studies focus on using permanent teeth for tooth-derived graft production, while recent studies have also demonstrated the effectiveness of using deciduous teeth in this field.^18,19^

 In the next step, which is tooth grinding, a hammer is used in Bone Maker and Vacuasonic devices to crush the teeth before milling. The crushed teeth are then ground with a high-speed or low-speed mill. Minetti et al.^31^ showed that a significant portion of the tooth and graft material is preserved when a low-speed grinder is used.^31^

 Since proteins can promote osteoinduction, the protein content of teeth is a key factor. Protein constitutes approximately 3.5‒4% of the tooth weight, consisting of autologous collagenated hydroxyapatite and autologous Type 1 collagen. Therefore, performing detoxification, demineralization, and trituration without careful consideration may result in a graft material that possesses only osteoconductive properties.^17^

## Shape of the Produced Material

 Tooth grafts can be produced in two forms: granular or block. In the granular form, the particle size of the granules produced by the existing devices ranges from 300 to 1200 μm (Table 1). Smaller particles facilitate bone resorption and remodeling, whereas larger particles help prevent rapid bone resorption.^32^ In the block form, tooth grafts are indicated for extraction socket preservation, bone defect reconstruction, and ridge augmentation with simultaneous implantation.^33,34^

## Demineralization of the Dentin Matrix (DDM)

 In the DDM process, highly crystalline inorganic components are removed.^35,36^ However, growth factors, collagen, non-collagenous proteins, and residual calcium phosphate are present.^36^ The demineralization process opens the dentinal tubules, releasing growth factors that promote tissue repair and regeneration. Growth factor β_1_ (TGF-β_1_), BMPs, vascular endothelial growth factors (VEGF), fibroblast growth factor-2 (FGF-2), platelet-derived growth factor (PDGF), and insulin-like growth factor-1 (IGF-1) are preserved and activated during the DDM process.^37-41^ Therefore, it is osteoinductive and osteoconductive.^42^

 Although there is no standardized protocol for DDM preparation,^35^ the general steps are as follows: soft tissues, caries, filling materials, and the enamel portion of extracted teeth should be removed, and the dentin should be ground into particles or cut into blocks.

 Multiple types of materials, such as chloroform/methanol or sodium hydroxide, can be used for defatting.^43^ Various acids and methods have been used in studies to demineralize dentin (Table 2). The most common demineralizing agents used are hydrochloric acid (HCl) and ethylenediaminetetraacetic acid (EDTA).^37,51-53^ Citric acid and nitric acid have also been utilized.^32,47,54^

**Table 2:** Outcomes and effectiveness of DDM

**Author**	**Type of the study**	**- Demineralizing Agent** **- Time**	**- Type of the particles** **- Size of the particles**	**Purpose**	**Experimental Groups**	**Outcomes**
Murata et al. (2022)^27^	Report of 20 years follow up a case	- 0.6 N HCL - 15 hours	- Powder - NM	Sinus floor augmentation	APDDM	Successful bone formation
Koga et al. (2016)^32^	Animal study (Rat)	- HNO3 2% - NM	- Powder - Three groups of: 200, 500, and 1000 μm	Calvaria defect fill	UDDm PDDM CDDM	PDDM with larger particle size showed better results.
Calvo-Guirado et al. (2023)^44^	Animal study (Rabbit)	- EDTA 10% - 2 min	- Powder - 300 – 1200 μm	Calvaria sites fill	No treatment BTCP PDDM	Higher bone regeneration was observed in the PDDM group.
De Oliveira et al. (2013)^45^	Animal study (Rat)	- EDTA 10% - 3 months	- Powder - NM	Socket preservation	No treatment HDDM	HDDM acts as a scaffold for osteoblast differentiation and represents new bone formation
Kim et al. (2021)^46^	Animal study (Rabbit)	- H2O2 - 1-2 min	- Powder - NM	Calvaria sites fill	DDM HDDM + PRF HDDM + rhBMP-2	DDM + rhBMP-2 represented higher bone formation
Minamizato et al. (2017)^47^	Cohort	- 2% HNO3 - 10 min	- Powder - 400-800 μm	Sinus floor augmentation and alveolar bone formation	APDDM	Bone regeneration observed in most of the cases.
Pang et al. (2017)^48^	RCT	- HCL - NM	- Powder - 300-800 μm	Alveolar bone augmentation for implant	ADDM Bio-Oss	ADDM was as effective as Bio-Oss for vertical augmentation of socket extraction. Similar efficacy in both groups.
Xu et al. (2018)^49^	Animal study (Rabbit)	- Hydrochloride 0.6 N - 15 min	- Powder - 0.8- 1.0 mm	Maxillary sinus sites fill	HDDM Bio- Oss	Significant higher new bone formation in the hDDM group
Yang et al. (2023)^50^	RCT	- 3% HCl - NM	- Powder - 425 to 1200	Alveolar ridge preservation	APDDM Spontaneous healing	Significant higher increased obtained in APDDM in the height of the central bone.

NM: not mentioned; ADDM: autogenous demineralized dentin matrix; APDDM: autogenous partial demineralized dentin matrix; BTCP: beta tricalcium phosphate; HDDM: human demineralized dentin matrix; rhBMP-2: recombinant human bone morphogenetic protein-2; PDDM: partial demineralized dentin matrix; UDDM: unmineralized dentin matrix; CDDM: complete demineralized dentin matrix; EDTA: ethylenediaminetetraacetic acid

 The degree of demineralization and particle size have been reported to influence the extent of bone regeneration; however, their effects appear to be dependent on the specific experimental or clinical context.^32,55^

 Koga et al.^32^ reported that partial dentin demineralization matrix (PDDM), defined as approximately 70% demineralized dentin, required 20 minutes of demineralizing for 1000-μm particles, while complete dentin demineralization (CDDM) required 180 minutes. Greater bone formation was observed in PDDM compared to CDDM; however, this difference was closely related to the degree of demineralization and particle size, as faster resorption of CDDM particles, particularly smaller ones, appeared to precede new bone formation.^32^ These findings indicate that the reported superiority of PDDM over CDDM is not absolute but strongly dependent on the demineralization protocol, particle size, and resorption kinetics.

 Although Koga et al.^32^ reported greater bone formation with dentin particle sizes of around 1000 μm in a rat calvarial defect model using partially demineralized dentin matrix, particle size alone does not appear to be a universally determining factor for bone regeneration.^32^ Other studies have reported favorable outcomes using smaller particle ranges, typically between 400 and 800 μm, particularly in clinical applications where partially demineralized dentin was combined with adjunctive materials such as platelet concentrates.^47^ These findings suggest that the effect of particle size on bone regeneration is context-dependent and may be influenced by factors such as the degree of demineralization, clinical versus experimental setting, and the use of biologically active adjuncts, rather than particle size alone.^32,47,56^

## Application of DDM

 Several animal studies used DDM to promote bone formation in rat and rabbit calvarial defects, rabbit parietal defects, and mini-pig cranial defects.^43-45,57^ DDM has the potential to support both osteochondral and bone regeneration.^43^

 The Korea Tooth Bank and Hospital Tooth Bank (HTB) process stored patients’ teeth into DDM for use as bone material.^13^ Several studies have demonstrated the safety and effectiveness of DDM as a bone substitute (Table 2). Socket preservation, maxillary sinus floor augmentation, and alveolar ridge augmentation are some indications of DDM.

## Heterogeneous Tooth Graft

 One major drawback of using a patient’s own teeth to produce AutoDG is the limited availability of graft material. To overcome this limitation, heterogeneous tooth graft approaches have been explored in preclinical studies. However, most available evidence is derived from animal models, and these approaches remain experimental, with unresolved ethical, immunological, and regulatory concerns. The main challenge in using heterogeneous teeth for graft preparation is preventing immune rejection. Current protocols attempt to reduce antigenicity mainly through extensive deproteinization and sterilization; however, complete elimination of immunologic risk has not been conclusively demonstrated, particularly beyond animal models.^45,58^

 Unfortunately, there is no standardized method for producing heterogeneous tooth grafts. In the following section, some of the main methods of preparing bone from heterogeneous teeth are given. It seems that other articles have also used the same techniques or modified them.^45,56^ Although the use of heterogeneous teeth to produce bone graft materials remains controversial from an ethical and technical standpoint, some researchers have employed this approach.

**Figure 1:**
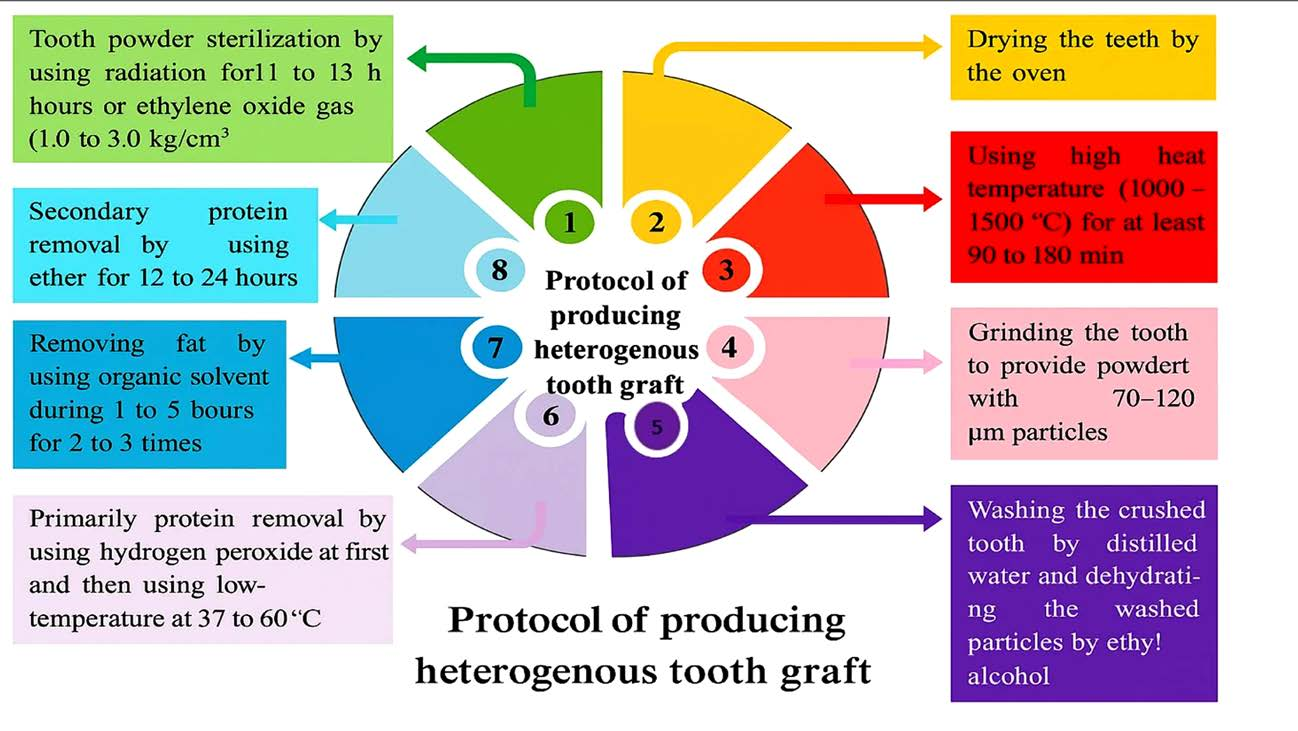
Method of producing heterogeneous graft material based on a South Korea Google patent

 South Korea published a Google patent (publication number: KR 1011139337B1 and available at https://patents.google.com/patent/KR101139337B1/en), describing the production of graft material from heterogeneous teeth, which is summarized in Figure 1. In this method, the use of high temperatures (1000‒1500°C) to eliminate immune rejection factors alters the fine surface structure. High temperatures increase crystalline apatite formation, cause loss of carbonic acid, and reduce bone conductivity.

 Calvo-Guirado et al.^44^ used human sterilized tooth particles to promote bone formation in rabbit calvarial defects, employing various protocols. The tooth preparation process involved extracting teeth from 10 patients, separating the crowns and roots, removing soft tissues, cleaning the roots, crushing them into particles ranging from 300 to 1200 μm, immersing the particles in basic alcohol cleanser consisting 20% ethanol and 0.5 M sodium hydroxide for 2 min, treating them with 10% EDTA for partial demineralization, washing them with phosphate-buffered saline solution twice for 5 min, and sterilizing them in an autoclave. Despite the absence of high-temperature heat in their protocol, no cases of immune rejection were reported. However, due to the diversity of methods used, further studies are needed.

 In another approach, Moraes et al.^58^ removed the cementum, dental pulp, and pre-dentin, and obtained 500-μm particles from heterogeneous teeth using a grinder. Centrifugation was performed for chemical separation. The resulting human dentin matrix was first moistened with deionized water for 5 hours. Then, it was mechanically cleaned for 20 minutes every hour using an ultrasonic cleanser. Further procedures were undertaken to generate DDM as follows: the teeth were immersed in a 17% EDTA solution for 5 minutes, then washed with deionized water for 10 minutes in an ultrasonic cleaner. They were subsequently exposed to 10% EDTA for 5 minutes, followed by another 10-minute wash with deionized water in an ultrasonic cleaner. The samples were then exposed to a 5% EDTA demineralizing solution for 10 minutes, followed by a final 10-minute wash with deionized water in an ultrasonic cleaner, and then dried. Immediately after this procedure, HDDM was sterilized by gamma irradiation for 18 hours and 58 minutes at 27°C with a 14.5-kGy dose.^16^ The sterilized material was stored in cryopreservation at -80°C.

 It should be emphasized that the sterilization and demineralization protocols described, including EDTA treatment, gamma irradiation, autoclaving, or heat processing, have been applied within experimental settings and animal models, and are not currently standardized or approved for routine clinical use. Moreover, these processing methods substantially alter the physicochemical properties of dentin-derived materials. Increased crystallinity and reduced resorption, as reported in animal studies, may enhance space maintenance but can also compromise biological remodeling and osteoconductivity.^44^ Therefore, although heterogeneous tooth-derived grafts have demonstrated osteoconductive and osteoinductive potential in animal models, their long-term safety, immunologic behavior, ethical acceptability, and regulatory status remain insufficiently defined. Further standardized investigations and controlled clinical studies are required before clinical translation can be considered.

## Tooth-derived Grafts in Comparison to Other Bone Graft Materials

 Tooth-derived grafts can be used alone or combined with other bone graft materials, such as bovine bone, Bio-Oss, PRF, PRP, and their modifications. Multiple studies have compared the efficacy of tooth grafts with other bone graft materials, summarized in Table 3.

**Table 3:** Comparison of tooth graft efficacy with other bone graft materials

**First author (Year)**	**Type of the study**	**Cases (T/ C)**	**Sex (F/M)**	**Aim**	**Bone graft material (T/C)**	**Follow-up**	**Main outcomes**
Santos et al. (2021)^22^	RCT	36/36 patients	31/21	Alveolar ridge preservation	Test: DDM + Bio-Guide membrane Control: Bio-Oss + Bio- Guide membrane	18 months	New bone formation, implant stability quotient, residual graft material
Kızıldag et al. (2020)^26^	Animal study	18 rabbits	0/18	Peri- implant bone defects reconstruction	T_1_: ATBG T_2_: ATBG + PRF C: No treatment	28 days	New bone formation and bone to implant contact (Histomorphometry evaluation)
Kim et al. (2021)^46^	Animal study	12 rabbits	0/12	Calvaria bone defects reconstruction	Group 1: DDM Group 2: DDM + PRF Group 3: DDM + rhBMP-2 Group 4: Control group with no treatment	2, 4, 8 weeks	New bone formation (Histopathologic and histomorphometry evaluation)
Pang et al. (2017)^48^		15/9	13/11	Augmentation of alveolar bone defects	Test: DDM Control: Bio-OSS	6 months	Sinus height, new bone formation, implant stability quotient
Moraes et al. (2022)^58^	Animal Study	20 rabbits	_	Alveolar ridge preservation	Group 1: Blood clot Group 2: Autogenous bone Group 3: Bovine- derived xenograft Group 4: Demineralized Human dentin matrix	7, 14, 28 days	New bone formation (Histopathologic and Microtomography volumetric evaluation)
Abdelraheim et al. (2023)^59^	RCT	16 patients	8/8	Alveolar ridge preservation	T: PDDM block C: A-PRF ^+^ plug	4 months	Increase in width and height of the crestal bone
Alrmali et al. (2023)^60^	Case series	26 patients	_	4 socket preservation cases, 5 cases of guided tissue regeneration, 5 cases of guided bone regeneration (GBR), 10 cases of sinus augmentation procedures, 2 immediate placement implants, and 2 socket shields	ATBG + PRF	32 months	New bone formation (Histologic analysis)
Andrade et al. (2020)^61^	Pilot clinical study	10 extraction sockets	_	Alveolar ridge preservation	ATBG + L- PRF	4, 5, 6 months	New bone formation (Histopathologic and histomorphometry evaluation)
Jun et al. (2014)^62^		19/19	14/24	Maxillary sinus bone graft	Test: DDM Control: Bio-OSS	4 months	Sinus height, new bone formation, implant stability quotient
Van Orten et al. (2022)^63^	Case series	7 patients	2/5	Socket preservation	Tooth-derived granules + i-PRF		Horizontal bone regeneration and histological evaluation
Vares et al. (2022)^64^	Case report	1	0/1	Traumatic mandibular cyst defect reconstruction	ATBG + A- PRF	7 months	Bone remodeling (CBCT evaluation)

T: test group; C: control group; F: female; M: male; PDDM: partial dentin demineralized matrix; A-PRF: advanced platelet-rich fibrin, I-PRF: injectable platelet-rich fibrin; rhBMP-2: recombinant human bone morphogenetic protein-2; PRF: platelet-rich fibrin

###  Bovine Bone

 Jun et al.^62^ compared the use of autogenous tooth-derived graft material (AutoDG) with Bio-Oss in sinus bone grafts, reporting no differences in bone density, bone height, new bone formation, residual graft material, or bone marrow space. However, trabecular (P = 0.006) and osteoid (P = 0.025) thickness were significantly higher in the AutoDG group.

 Pang et al.^48^ evaluated the efficacy of AutoDG versus Bio-Oss for alveolar bone augmentation, reporting that although the vertical dimension of the alveolar bone and new bone formation increased more in the Bio-Oss group, there was no significant difference between the two groups.

 Santos et al.^22^ used a tooth graft for ridge preservation and reported no significant clinical or radiographic outcomes compared to Bio-Oss.

 A systematic meta-analysis comparing the osteogenic effectiveness of tooth grafts and Bio-Oss in bone augmentation procedures found no significant difference in new bone formation between the two materials. The review represented that tooth graft is just as effective as Bio-Oss for bone augmentation.^65^

 Moraes et al.^58^ conducted an experimental study to compare the efficacy of bovine bone and heterogeneous human DDM for alveolar ridge preservation. The results showed that both the bovine bone and DDM groups exhibited similar outcomes. The DDM group demonstrated new bone formation and slow resorption. Additionally, the study concluded that DDM can help preserve the alveolar ridge after tooth extraction.

###  Platelet-rich Fibrin (PRF)

 Kizildağ et al.^66^ evaluated new bone formation using a tooth-derived graft with PRF. Bone augmentation and bone density were significantly higher in the group that received a tooth-derived graft combined with PRF compared to the group receiving only the tooth-derived graft.

 Khunger et al.^67^ reported that the use of a tooth-derived graft material mixed with PRF for immediate dental implant placement resulted in increased new bone formation and reduced alveolar bone resorption.

 In an RCT by Abdelraheim et al.^59^, the mean value of bone density was significantly higher in the group that combined PRF with tooth graft material, compared to the control group that did not receive PRF.

 An animal study on the effect of tooth-derived graft in combination with PRF in peri-implant defects showed that new bone formation was significantly higher in the ATBG (Autogenous Tooth Bone Graft) + PRF group compared to the ATBG group or the control group.^26^ In contrast, Kim et al.^46^ did not find a significantly higher amount of bone formation between the DDM group and the DDM + PRF group. In contrast, the DDM combined with recombinant human bone morphogenetic protein-2 (rhBMP-2) group showed a significantly higher degree of bone formation.

 Alrmali et al.^60^ used ATBG mixed with PRF and demonstrated that this material can be an effective alternative to traditional bone graft materials in GBR procedures and maxillary sinus augmentation (sinus elevation) operations.

 Although multiple studies have reported improved bone formation when PRF is combined with tooth-derived graft materials, the findings are inconsistent, with some studies not showing a significant additional benefit.

###  Advanced Platelet-rich Fibrin (A-PRF)

 Gowda et al.^68^ reported that the application of PDDM is more efficient for socket preservation than advanced platelet-rich fibrin. The midbuccal and palatal crestal height, as well as the alveolar ridge width, were significantly higher in the PDDM group. In contrast to the control group, the test group did not show a significant difference in the resorption of alveolar ridge width.

 Vares et al.^64^ found that grafting a traumatic mandibular defect with ATBG mixed with A-PRF is an appropriate bone graft biomaterial to restore even sizeable bone defects.

###  Injectable Platelet-rich Fibrin (i-PRF)

 van Orten et al.^63^ reported that a combination of i-PRF with tooth-derived granules is a valuable method for socket preservation, resulting in high-quality bone.

###  Leukocyte-platelet-rich Fibrin (L-PRF)

 Andrade et al.^61^ used a mixture of ATBG and L-PRF in extraction sockets, reporting preservation or even enhancement of the vertical and horizontal dimensions of the alveolar ridge.

###  Platelet-rich Plasma (PRP)

 Kim et al.^69^ used PRP along with autogenous tooth graft blocks for sinus bone augmentation. They reported that after a 2-year follow-up, there was no significant difference in residual alveolar bone height or new bone volume between the PRP and control groups.

###  Beta Tricalcium Phosphate (BTCP)

 An animal study comparing tooth graft materials and the BTCP biomaterial reported that the tooth graft materials significantly increased bone formation to a greater extent than the BTCP material.^44^

 Overall, available evidence suggests that tooth-derived grafts may yield clinical and histological outcomes comparable to those of other bone graft materials. The clinical translation of tooth-derived grafts offers potential for various applications in implant dentistry, alveolar ridge preservation, and maxillofacial reconstruction. However, the absence of standardized preparation protocols, heterogeneity among studies, small sample sizes, and the limited number of well-designed long-term clinical trials remain significant challenges.

## Discussion

 The clinical and histological outcomes reported across multiple studies consistently indicate that dentin-based grafts provide bone regeneration comparable to that of xenografts and alloplasts, with the added advantage of being autogenous and eliminating disease transmission risks.^7,9,22,48,62^ The intrinsic presence of bioactive molecules such as BMPs, TGF-β, and IGFs further enhances their osteoinductive potential.^13,37-41^

 Studies have demonstrated that partial demineralization preserves calcium phosphate for structural stability while allowing the release of embedded growth factors, leading to superior osteogenic activity compared with either fully mineralized or completely demineralized dentin.^32,47,50^ Similarly, the particle size of dentin granules influences the rate of resorption and new bone formation. Smaller particles accelerate remodeling, whereas larger particles maintain graft volume and mechanical stability over time.^32,55^

 The composition of processed material also plays a pivotal role. While some authors exclude enamel due to its dense crystalline structure and limited resorbability, others argue that the inclusion of enamel particles can enhance space maintenance and graft volume.^10,17^ The cementum layer, when preserved, has shown histological integration with surrounding bone, suggesting a potential role in osteoconduction.^27-29^ Despite these findings, a universal consensus on the optimal combination of dentin, enamel, and cementum components remains lacking, and variations among existing commercial processing devices complicate standardization.

 The incorporation of platelet concentrates appears to significantly improve the biological response and bone density when combined with tooth-derived grafts.^60-63^ PRF provides autologous growth factors and fibrin scaffolding that synergize with dentin-derived bioactive proteins, accelerating angiogenesis and bone remodeling. However, the degree of improvement varies across studies and depends on the PRF preparation method and the type of graft used.^46,59,67^ Collectively, these findings suggest that optimizing the physicochemical parameters of tooth-derived grafts—particularly balancing structural stability with the controlled release of bioactive molecules—may play a more critical role in clinical performance than the specific choice of processing device or preparation technique.

 Compared with bovine xenografts such as Bio-Oss, tooth-derived grafts show comparable or superior performance in terms of new bone formation, trabecular thickness, and osteoid maturation, while avoiding the limitations of xenogeneic materials such as slow resorption or potential immunogenicity.^22,48,62,65^ In addition, clinical trials have demonstrated that tooth-derived grafts can be used effectively for sinus augmentation, ridge preservation, and peri-implant bone regeneration without significant adverse events.^44,50,64^

 While short-term outcomes reported in the available literature are generally encouraging, important questions regarding long-term degradation kinetics and clinical predictability remain insufficiently addressed. The relative resorption rate of dentin compared to native bone, the long-term fate of enamel-containing graft particles, and extended implant survival in dentin-grafted sites have not yet been comprehensively evaluated. In addition, complication profiles—including infection, membrane exposure, and volumetric stability—have not been systematically reported across standardized follow-up periods. These gaps highlight the need for longer-term and methodologically consistent investigations.

 Within the broader landscape of contemporary regenerative dentistry, several alternative biomaterial strategies have been developed, including bioactive glasses, customized allograft blocks, 3D-printed scaffolds, growth-factor-enhanced biomaterials, and cell-based constructs. These approaches differ in terms of biological activity, structural properties, and translational readiness. Tooth-derived grafts represent an autogenous and biologically derived alternative within this spectrum. However, direct comparative data between dentin-based grafts and these modern regenerative technologies remain limited, and clear clinical superiority has not been established. Future investigations should aim to position dentin-derived materials within this broader regenerative framework through standardized comparative methodologies.

 Despite the promising findings reported in the literature, several important limitations must be acknowledged. The current body of evidence is characterized by substantial methodological heterogeneity, including variability in dentin processing protocols such as demineralization duration, acid concentration, sterilization techniques, and particle preparation methods. These inconsistencies may directly influence material properties and reported clinical outcomes. Furthermore, most available studies are limited by small sample sizes, short follow-up durations, and diverse study designs.

 Given the emerging nature of this field, high-level RCTs remain limited, and a considerable proportion of the evidence is derived from preclinical investigations or pilot clinical studies. As a result, definitive conclusions regarding long-term clinical superiority or standardized clinical indications cannot yet be established. In addition, long-term resorption kinetics, degradation behavior relative to native bone, implant survival in dentin-grafted sites, and the potential influence of systemic conditions remain insufficiently clarified.

 It is also important to note that, as a comprehensive narrative review synthesizing heterogeneous evidence, formal quantitative weighting of studies was not performed. This should be interpreted as a methodological scope decision rather than an attempt to equate different levels of evidence. The heterogeneity of study designs and outcome measures was explicitly taken into account in the interpretation of the findings.

 From a regulatory and translational perspective, the use of heterogeneous or extensively processed tooth grafts continues to raise ethical and immunologic considerations. Although current preparation protocols aim to reduce antigenicity, comprehensive safety validation and regulatory standardization are still required before widespread clinical implementation.

 Future investigations should prioritize well-designed multicenter randomized clinical trials with standardized preparation protocols and extended follow-up periods to better define long-term success rates of implants placed in tooth-derived grafted sites. Comparative studies evaluating different processing devices and preparation strategies are also warranted. Additionally, integrating advanced biotechnological approaches—such as recombinant growth factors, bioactive molecules, and stem cell-based therapies—may further expand the regenerative potential of these biomaterials. To facilitate regulatory acceptance and clinical translation, establishing GMP-compliant manufacturing workflows and completing ISO 10993 biocompatibility testing will be essential prerequisites.

## Conclusion

 Tooth-derived grafts remain an attractive option for bone regeneration in dentistry due to their biocompatibility, osteoconductive and osteoinductive properties, and low immunogenicity. Studies have shown that tooth-derived grafts are comparable in quality to commonly used bone graft materials. When combined with platelet factors such as PRF, they can enhance bone regeneration outcomes. However, additional controlled clinical studies are required to provide a more comprehensive comparison with other bone graft materials. It is also recommended that, to enable the broader use of tooth-derived grafts, future research should focus on developing a specific protocol for producing DDM.

## Competing Interests

 The authors declare that they have no competing interests regarding authorship and/or publications of this paper.

## Data Availability

 The data that support the findings of this study are available from the corresponding author upon reasonable request.

## Ethical Approval

 The Protocol was registered with and approved by the Ethics Committee of the Medical University of Isfahan (IR.MUI.DHMT.REC.1403.123).
